# Temporary Redundant Transmission Mechanism for SCTP Multihomed Hosts

**DOI:** 10.1155/2015/158697

**Published:** 2015-01-18

**Authors:** D. Mohana Geetha, S. K. Muthusundar, M. Subramaniam, Kathirvel Ayyaswamy

**Affiliations:** ^1^Department of Electronics and Communication Engineering, S. A. Engineering College, Chennai, Tamil Nadu 600077, India; ^2^Department of CSE, Sri Muthukumaran Institute of Technology, Chennai, Tamil Nadu 600069, India; ^3^Department of Computer Science and Engineering, S. A. Engineering College, Anna University, Chennai, Tamil Nadu 600077, India; ^4^Department of Information Technology, Anand Institute of Higher Technology, Anna University, Chennai, Tamil Nadu 603103, India

## Abstract

In SCTP's Concurrent Multipath Transfer, if data is sent to the destined IP(s) without knowledge of the paths condition, packets may be lost or delayed. This is because of the bursty nature of IP traffic and physical damage to the network. To offset these problems, network path status is examined using our new mechanism Multipath State Aware Concurrent Multipath Transfer using redundant transmission (MSACMT-RTv2). Here the status of multiple paths is analyzed, initially and periodically thereafter transmitted. After examination, paths priority is assigned before transmission. One path is temporarily employed as redundant path for the failure-expected path (FEP); this redundant path is used for transmitting redundant data. At the end of predefined period, reliability of the FEP is confirmed. If FEP is ensured to be reliable, temporary path is transformed into normal CMT path. MSACMT-RTv2 algorithm is simulated using the Delaware University ns-2 SCTP/CMT module (ns-2; V2.29). We present and discuss MSACMT-RTv2 performance in asymmetric path delay and with finite receiver buffer (rbuf) size. We extended our experiment to test robustness of this algorithm and inferred exhaustive result. It is inferred that our algorithm outperforms better in terms of increasing the throughput and reducing the latency than existing system.

## 1. Introduction

The Stream Control Transmission Protocol (SCTP) can be used to transmit real-time traffic over IPv4/IPv6 networks. SCTP has enhancing features of Multistreaming and Multihoming. SCTP is capable of transmitting different types of data simultaneously on a single stream with its Multistreaming feature to its destination through multiple paths as Multihoming supports multiple IP addresses within its single association. Multihoming can be expected [1] to become the rule rather than the exception in the near future as cheaper network interfaces and internet access motivate content providers to have simultaneous connectivity through multiple ISPs and more home users install wired and wireless connection for added flexibility and fault tolerance. A host is multihomed [2] if it can be addressed by multiple IP addresses, as in the case when the host has multiple network interfaces.

Multihoming, magnificence of having more network interfaces ensures better performance and 100% availability for our today's critical Internet. Increase in number of interfaces helps us to get connected with different ISPs assuring the network redundancy. Large enterprises, campuses, and data centers have traditionally used Multihoming to multiple ISPs as a way of ensuring continued operation during connectivity outages or other ISP failures. Multihoming is leveraged for improving WAN performance, lowering bandwidth costs, and enables 100% network uptime.

From our experimental analysis, CMT-PF works well for the dual-homed system with less frequent path failures. When using more than two paths and frequent failures on those paths, the performance affirms worst [3, 4]; this is because when the retransmission timer is larger, the sender has to wait for expiry of retransmission timer, for retransmitting in active path(s). This minor delay in each failure accumulates and results in higher latency and thereby degrades the overall throughput. Therefore soundness of our proposal is effective for the systems having more than two paths and with increased frequency of failures. In Section 2 we review the earlier works. In Section 3, Algorithm 1 we look into revised version of MSACMT-RT algorithm named as MSACMT-RTv2 and Section 3.1 discuss validation of algorithm and in Section 3.2 discuss network simulation topology. The performance is evaluated in Section 4 and in Section 5 we conclude our work.

## 2. Literature Study

We know that SCTP supports Multihoming; Concurrent Multipath Transfer (CMT) is the concurrent transfer of new data from a source to a destination host via two or more end-to-end paths [5]. CMT between multihomed hosts increases the application's throughput [1, 6]. SCTP's Multihoming feature having multiple interfaces with multiple IPs allows data to be transmitted through multiple interfaces; in case of failure of the primary path, data is automatically transmitted through its alternate path (IP) [2, 5]. However retransmission in alternate path due to failure in primary may not be suitable in wireless environments such as Future Combat Systems (FCS), since wireless links introduce an additional loss factor: noisy channels, and therefore unexpectedly performed worst under this condition and often degraded performance [7].

The primary reasons found for the degradation in performance are related with lack of sufficient data flow (traffic) underutilization of bandwidth on alternate paths [7]. As discussed by Stewart et al. [5] default Path.Max.Retransmit (PMR), based on a network's loss rate which translates to ≈63 s (6 consecutive timeouts for failure detection). Adopting the value PMR = 5 (threshold), whenever the value goes beyond the set threshold, the sender retransmits in alternate path.

Caro et al. [1] and Iyengar et al. [8] explored five retransmission policies for CMT and their overall analysis revealed that RTXLOSSRATE, RTX-SSTHRESH, and RTX-CWND outperforms better. Their analysis of CMT in SCTP Multihoming using finite and infinite receive buffer (rbuf) also resulted in the fact that RTX-SSTHRESH and RTX-CWND retransmission policies alleviated some of the throughput degradation by reducing the rbuf blocking problem [9]. Of the practical loss rate based policies (RTX-CWND and RTX-SSTHRESH), RTX-SSTHRESH was chosen as the best CMT's retransmission policy from their investigation under different end-to-end delays [9].

Natarajan et al. [10] included Potentially Failed (PF) in CMT and demonstrated the CMT-PF's abilities that avoided back-to-back timeouts on data improving its performance over CMT's when the paths have asymmetric loss rates. Liu et al. [11] further alleviated some of the throughput degradation caused by rbuf blocking problem in SCTP-CMT by combining the parameters and suggested compound parameter retransmission policy (Rtx-CSL policy) and improved the goodput under infinite rbuf only. However to achieve faster yet robust failure detection, Caro [12] argues varying Path.Max.Retransmit (PMR) based on a network's loss rate and suggested PMR = 3 for the Internet. Also, a tradeoff exists for deciding PMR value—a lower value reduces rbuf blocking but increases the chances of spurious failure detection, whereas a higher PMR increases rbuf blocking and reduces spurious failure detection in a wide range of environments [13].

Trong et al. [14] achieved good throughput for single radio multichannel multipath wireless mesh networks, by utilizing scheduling availability with only one wireless card in single path and omitted CMT. Our work rests on the foundations of excellent system already proposed in [15–18], so called Non-Renegeable SACK (NR-SACK). This NR-SACK significantly improved the CMT transport's performance over dissimilar paths, as shown in [19–21], by allowing a sender to remove gap-acknowledged chunks from its sender buffer. We include the above discussed concept in our MSACMT-RTv2 (depicted in Section 3) mechanism for improving the overall performance of the system.

## 3. Multipath State Aware CMT-RTv2

The MSACMT-RT employs a redundant path (a.k.a. supporting path), for highest prioritized path. During transmission, in case of failure of this highest prioritized path, the same data carried by this redundant path will be delivered to the application, thereby preventing blocking of receiver buffer. But paths are prioritized from highest to least order, which is having least round trip time (RTT), largest congestion window (cwnd), and largest slow start threshold (ssthresh) and low loss rate (lossrate).

From the above assumptions, it is clear that a path with less priority is more prone to failure than the higher order prioritized path. Under this circumstance, employing *n*th prioritized path as redundant path for *n*-1st (this redundant path will act temporary) will function more effectively than assigning it for 1st prioritized path. Applying this basic logic as the first difference MSACMT-RTv2 differs from MSACMT-RT. The second difference is, after ensuring the reliability of FEP, subsequent cycle employs all paths for normal CMT. When this normal cycle successfully transfers ten transmissions, the control is transferred for including any failed path that has turned active and/or assigning new priority for subsequent transmission and converting the aforesaid redundant path to normal path.

Therefore this redundant path works only temporarily; hence forth we say redundant transmission is temporary (i.e., Temporary Redundant Transmission mechanism for SCTP multihomed host). These are the main two differences introduced and implemented in Steps 6 and 7 of the MSACMT-RT (MSACMT-RTv2), which improves the effectiveness considerably. The main advantage of this algorithm is that the path priorities are redefined after ten transmissions (in Algorithm 1, step 10); this enhances the performance further by rechecking the durability of all paths.

### 3.1. Determining the Review Period for Algorithm

In this section MSACMT-RTv2 review period is determined. By design one path has to be chosen as a redundant path for the path which is expected to face failure. A path is expected to face failure whose quality is relatively poor. The quality of paths is assessed based on parameters that the path has. The order of ascertaining is the path with, (i) Smallest Round-trip-time/Retransmission-timer-off (RTT/RTO), (ii) Largest congestion Window (cwnd), (iii) Largest Slow-start-Threshold (ssthresh), (iv) Smallest Loss-rate (lossrate) and in situations, if all the above values are similar for both paths, randomly order of priority is defined. The order of ascertainment is based on the theme and direction referred from [9, 11]; however RTT/RTO is the additional parameter prefixed in this evaluation.

*Note.* In practice several variations may happen as the following. (a) The path with smallest RTT/RTO may also have high lossrate or smallest cwnd and ssthressh. (b) The path with largest RTT/RTO may also have low lossrate or smallest cwnd and ssthressh. (c) The path with smallest RTT/RTO may also have smallest cwnd, ssthressh, and lossrate. (d) A high bandwidth path may also have a higher loss rate or a low bandwidth path may also have lower lossrate. And several other combinations other than discussed early would also occur; however as a rule-of-thumb in our experiment we stick asserting in the order as discussed above. Experiment referring to other combinations is left for our future research work.

#### 3.1.1. Analysis

**Figure 1:**
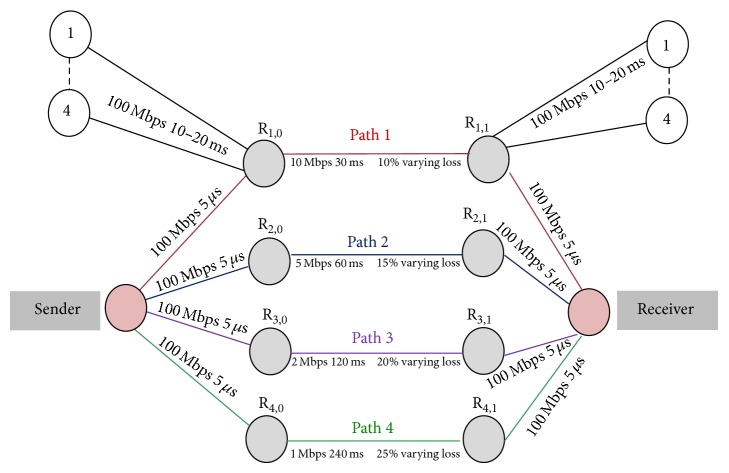
Simulation topology for validating algorithm.

We consider and simulate a typical network topology as shown in Figure 1, whereas the assigned parameters and network conditions are self-explanatory. The other edge nodes are single-homed and introduce cross traffic that instigates bursty periods of congestion and bursty congestion losses at each router. Each single-homed edge node has eight traffic generators (which are not shown in Figure 1). These cross traffic generators will be introduced between the routers R_1,0_ and R_1,1_ or R_2,0_ and R_2,1_ or R_3,0_ and R_3,1_ or R_4,0_ and R_4,1_ based on experimental requirement to introduce congestion on the respective transmission paths.

**Figure 2:**
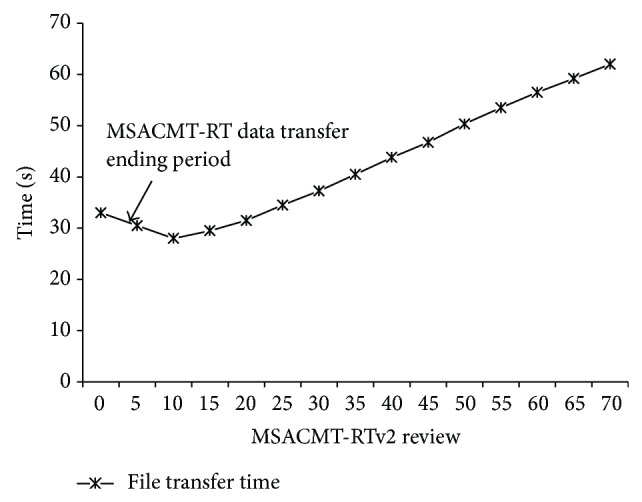
MSACMT-RTv2 reviews at number of successful transmissions.

In order to determine the aforesaid, simulation test is conducted transferring 20 MB file from the sender A to the receiver B using Path 1, Path 2, Path 3, and Path 4 concurrently with reference to the network simulation topology as shown in Figure 1.  Figure 2 shows the graph for various MSACMT-RTv2 cyclic values versus time taken for transferring 20 MB file. From Figure 2 it is inferred that the best performance is achieved if the iteration is reviewed after every ten successful transmissions which is said to be repetition cycles. The experiment is also conducted with varying file sizes, resulting in similar result. Hence it is concluded from this set of experiment to set the threshold to confirm the path durability as ten.

### 3.2. Network Simulation Topology

**Figure 3:**
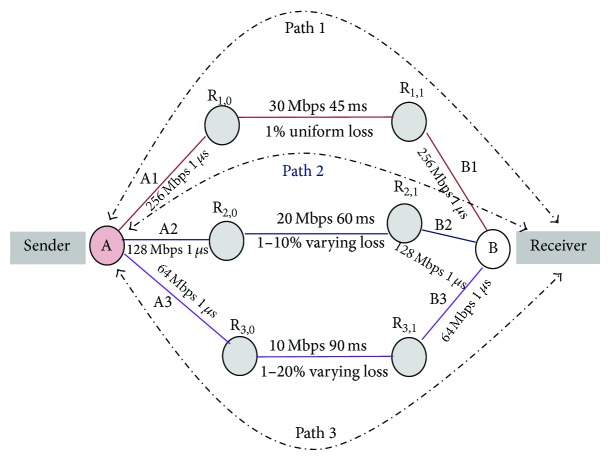
Network simulation topology.

In practical network environments services offered by different ISPs have various network characteristics and their characteristics also vary from time to time. Hence we consider and simulate a typical network topology as shown in Figure 3, whereas the assigned parameters and network conditions are self-explanatory. MSACMT-RTv2 receiver maintains a single rbuf, which is shared across the subassociation flows in an association. Irrespective of the layer at which Multipath transfer is performed, a similar shared buffer would exist at a receiver.

## 4. Performance Evaluation

In our simulation, we experimented by transferring various file sizes from the sender A to the receiver B using Paths 1, 2, and 3 concurrently using network simulation topology as shown in Figure 3. This file transfer uses a single streamed MSACMT-RTv2 association such that all data is delivered in sequence to the receiving application. The duplicate packets received by the receiver are discarded, but cumulative acknowledgement is sent in the path upon receiving any data packet.

### 4.1. Evaluation in Nonfailure Scenarios

We experimented transferring various file sizes, 20 MB, 30 MB, 40 MB, 50 MB, and 60 MB, respectively, where the initial congestion window is set to 2 MTU.  Table 1 shows the percentage of file transfer time advanced by MSACMT-RTv2 for various file sizes. Here it is understood that the larger the transfer file size, the larger the difference in file transfer time advanced. When application uses MSACMT-RTv2 for smaller size file transfer the difference is negligible.

### 4.2. Evaluation in Failure Scenarios

Failure scenarios are simulated by bringing down the bidirectional link between routers R_1,0_ and R_1,1_ or R_2,0_ and R_2,1_ or R_3,0_ and R_3,1_ at various intervals and the link is brought up at various intervals, as simulation topology in Figure 3.

#### 4.2.1. Evaluation of Path Failures at Regular Intervals

Failure induced simulation study is experimented by bringing down Path 2 during the file transfer; that is, the bidirectional link between routers R_2,0_ and R_2,1_ is brought down causing failures at regular intervals. We transfer file size of 60 MB; cwnd = 2 MTU from sender A to the receiver B using Path 1, Path 2, and Path 3. This one time failure is induced at 10th second and the link is brought up after 5 seconds. The file transfer time is calculated for MSACMT-RTv2 and MSACMT-RT. The time taken through MSACMT-RTv2 is 48.05 s, whereas MSACMT-RT is 55.02 s. Similarly two, three, four, and five time failures were introduced at regular intervals and their file transfer times are recorded as shown in Table 2. This table shows the percentage of time data transfer completed in advanced comparing with MSACMT-RT. MSACMT-RTv2 performs better during more number of failures.

#### 4.2.2. Evaluation of Path Failures at Irregular Intervals

We also evaluated the path failures at irregular interval of time. These failures were induced randomly during transmission which is assumed irregular intervals. Each failure persists for 5 s duration.

**Figure 4:**
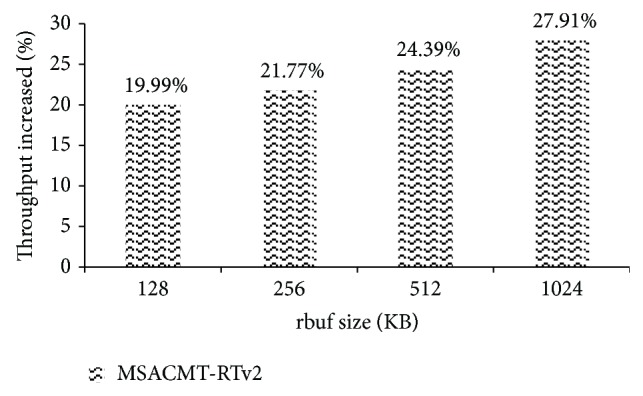
Percentage of throughput for various file sizes.

The file transfer time through MSACMT-RTv2 and by MSACMT-RT is shown as the percentage of data transfer time advanced by MSACMT-RTv2 in fourth column of Table 3. We had also experimented with different receiver buffer size of 128 KB and 256 KB and values are shown in Table 4. From Table 4 it is concluded that as number of failures increases MSACMT-RTv2 performs best. In Figure 4 the graph shows that the percentage of throughput increased shown for various receiver buffer values.

### 4.3. Robustness Analysis

Robustness test is used to ensure the degree to which a system or component can function correctly in the presence of invalid inputs or stressful environmental conditions. In this section we extend this emphasis on handling congestion [22, 23]. In this test we focus on studying the behavior of system during extreme traffic that is flooded in the transmission path, which is congestion in the network. This congestion is injected by sender into the different network path. The phenomena are observed while path experiencing symmetric and asymmetric loss conditions during failure and nonfailure scenarios.

#### 4.3.1. Experimental Investigations

**Figure 5:**
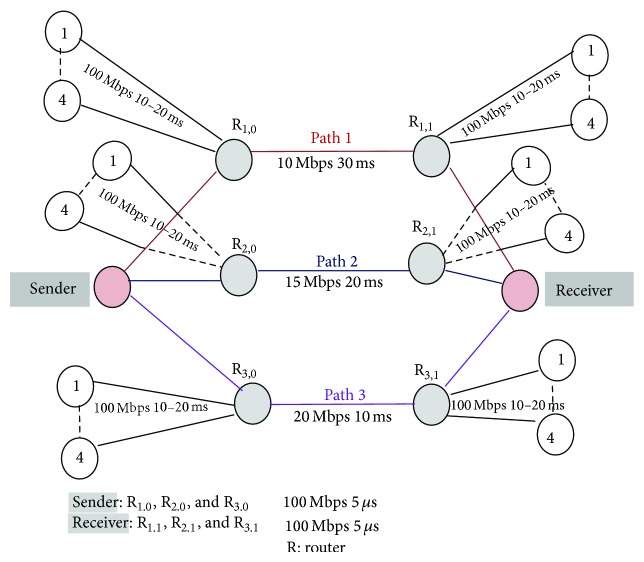
Simulation topology for asymmetric paths.

We take the simulation topology of triple-homed edge transceivers attached through routers R_(*x*,*y*)_ as shown in Figure 5 with cross traffic and path parameters, which is a more realistic loss model. The other edge nodes are single-homed and introduce cross traffic that instigates bursty periods of congestion and bursty congestion losses at the routers. Each single-homed edge node has eight traffic generators that will introduce cross traffic with a Pareto distribution. The cross traffic packet sizes are chosen to resemble the distribution found on the Internet: 50% are 44 B, 25% are 576 B, and 25% are 1500 B [24, 25]. The cross traffic flows start at random times during the initial 5 seconds of the simulation. After an initial warm-up period of 10 seconds, sender transmits a 40 MB file to receiver over Path 1, Path 2, and Path 3.

#### 4.3.2. Nonfailure Scenarios

For both MSACMT-RT and MSACMT-RTv2 flows, rbuf = 128 KB, PMR = 5, and loss rates are controlled by varying the cross traffic load. The result is a data transfer between sender and receiver, over a network with self-similar cross traffic, which resembles the observed nature of traffic on data networks [26]. The graphs in the subsequent discussions plot the average goodput (file size ÷ transfer time) of MSACMT-RT versus MSACMT-RTv2 with 5% error margin.

**Figure 6:**
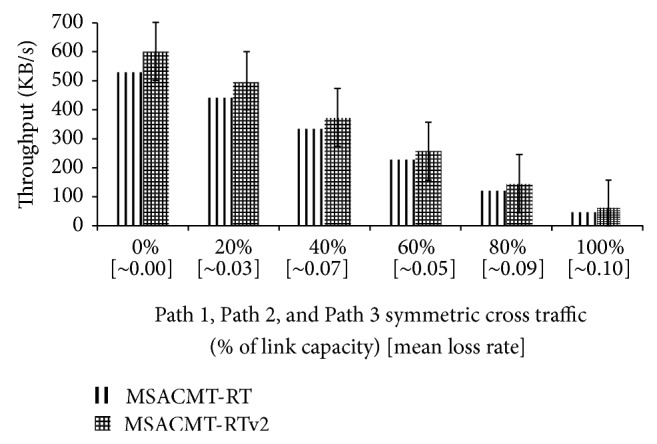
MSACMT-RT versus MSACMT-RTv2 during symmetric loss.

*(A) Symmetric Loss.* In the symmetric loss the aggregate cross traffic load on Path 1, Path 2, and Path 3 is similar. The symmetric cross traffic is varied from 0% to 100% of the core links bandwidth. In Figure 6, when the cross traffic load is high to the maximum of 100% the MSACMT-RT and MSACMT-RTv2 perform almost equal. When cross traffic load is low as 0% MSACMT-RTv2 performs better than MSACMT-RT. This is because redundant mechanism works well in high bandwidth by allowing more redundant packets to move without loss such that a more number of packets are reached in advance in the redundant path. As the cross traffic load increases along with increase in mean loss rate MSACMT-RTv2 performs better but not worse than MSACMT-RT.

**Figure 7:**
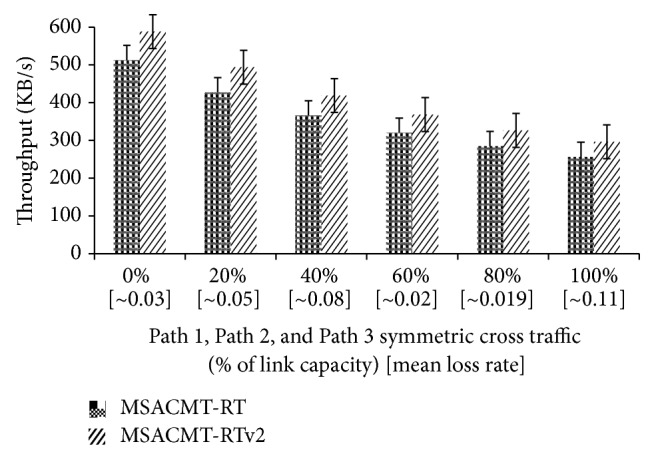
Asymmetric loss conditions, when Path 1 = 50% cross traffic.

**Figure 8:**
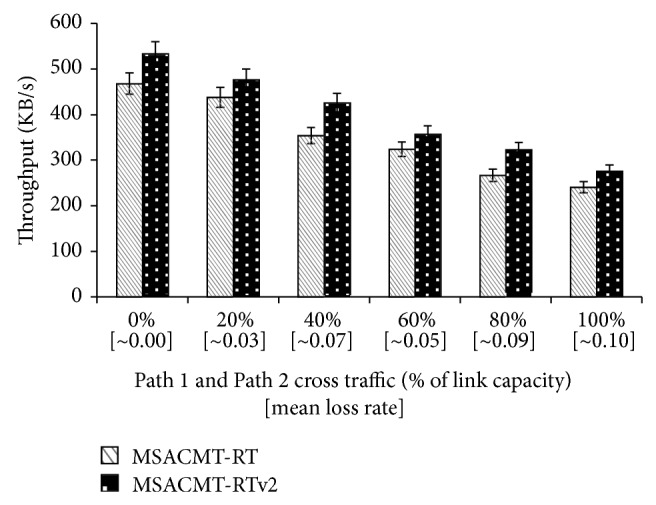
Asymmetric loss conditions: Path 3 = 50% cross traffic.

*(B) Asymmetric Loss.* In Figure 7, for asymmetric loss Path 1 cross traffic is set to 50% of the core link bandwidth; the *x*-axis label is self-explanatory. Since the available bandwidth on Path 2 and Path 3 has greater capacity and less delay induced cross traffic does not affect the relative timing of data transfer. But MSACMT-RT and MSACMT-RTv2 experience fewer throughputs as the induced cross traffic becomes higher. But the latency is low in MSACMT-RTv2 and the loss rate influences the average throughput. In Figure 8, Path 3 cross traffic is set to 50% of the core link bandwidth; the *x*-axis label is self-explanatory. There is variable delay up to 20 ms and 30 ms for Path 1 and Path 2 and more loss is experienced in this path; therefore throughput is relatively low when cross traffic at Path 1 and Path 2 is low. As the loss rate increases in high latency path, the probability that a sender experiences consecutive event in the path also increases.

#### 4.3.3. Failure Scenarios

Short-term failures (STF) are long enough for the sender to exercise back-to-back timeouts on the failed path, here in Path 3 (STF). In order to observe a prominent difference we elevate single short-term failures to multiple failures during the file transfer. Here we induce five times failure in Path 3 such that each failure lasts for 5 seconds and results were recorded.

**Figure 9:**
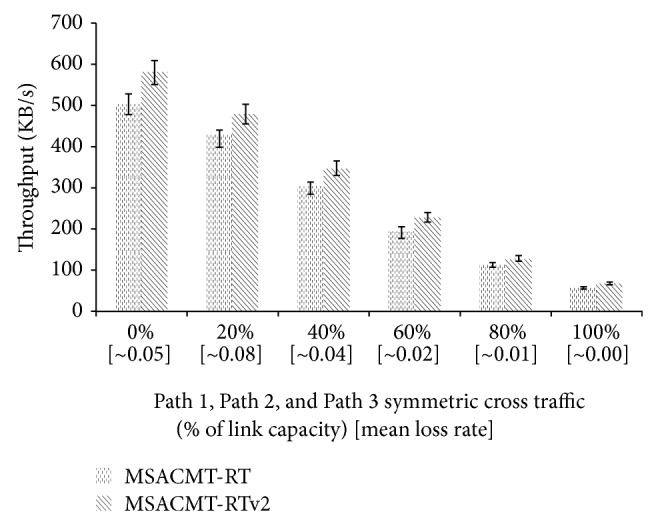
MSACMT-RT versus MSACMT-RTv2 during asymmetric loss.

*Symmetric and Asymmetric Loss.* In Figure 9, the symmetric loss the aggregate cross traffic load on all Paths is similar, except during the duration of failure in Path 3. When the cross traffic load is high to the maximum of 100% the MSACMT-RT and MSACMT-RTv2 perform almost equal. When cross traffic load is low as 0% MSACMT-RTv2 performs better than MSACMT-RT. The relative average throughput is greater in absence of uniform loss on all paths. As the cross traffic load increases along with increase in mean loss rate MSACMT-RTv2 performs better but not worse than MSACMT-RT.

**Figure 10:**
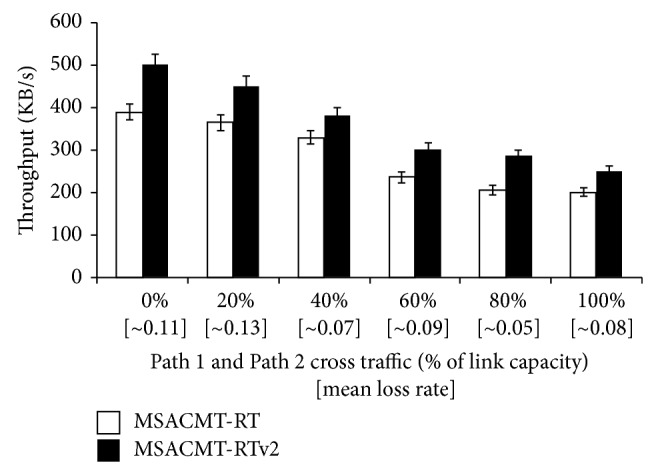
MSACMT-RT versus MSACMT-RTv2 during asymmetric loss conditions, when Path 3 = 50% cross traffic.

The average number of transmissions that MSACMT-RT and MSACMT-RTv2 take for transferring the file is shown in Table 5. In Figure 10, the graph is plotted for asymmetric losses. rbuf blocking depends on the frequency of loss events (loss-rate) and the duration of loss recovery. As the loss rate increases, the probability that a sender experiences consecutive timeout events on Path 3 increases, since Path 3 is caused to failure. But MSACMT-RT after the first timeout avoids data transmission on Path 3 except generate sending heartbeat until the path becomes active. But in MSACMT-RTv2 unless the loss occurs the transmission is not affected.

In Table 5, the number of transmission on each path consistently in MSACMT-RT but in the case of MSACMT-RTv2 as the path cross traffic load increased in Path 1 and Path 2, the highest priority path which assigned to have better quality functions more transmitting data's. Although Path 3 faces failures, the QoS parameter says to claim better for transmission; hence Path 3 has more number of data transmissions. In summary, referring to Table 6 shows that for each case the average throughput in Kbps for failure and nonfailure scenarios, MSACMT-RTv2 does not perform worse than MSACMT-RT during asymmetric path loss conditions. In fact MSACMT-RTv2 is a better transmission strategy than MSACMT-RT and performs better as the asymmetric path loss increases.

## 5. Conclusions and Future Work

MSCMT-RTv2 has been investigated in challenging scenarios with different sets of experiments on asymmetric path conditions during failure and nonfailure scenarios. Each of the experimental cases revealed that MSACMT-RTv2 algorithm agrees and performs better than MSACMT-RT. The robustness test result infers and ensures improving the overall throughput and reducing the latency in the presence of stressful (network congestion) conditions.

This experiment uses redundant path mechanism by assigning weakest path to function as redundant path for the weaker path. Weakest path is assigned as subsidiary path for weaker path; however both paths are stated to be weak with minor degree of variation. There is no guarantee that either of the paths will always function. After the commencement/resumption of transmission if both paths (redundant path and weaker path) fail, the situation will be regressed. Our experiment has not addressed this issue. MSACMT-RTv2 algorithm can be further reconstituted to encounter the above circumstances.

In addition dynamic review period may also be redefined to react based on network conditions. For example situation when paths quality remains unchanged for longer duration will face unnecessary delay in frequent path review (CSTRP = 10) while utilizing SCTP association for longer period.

## Figures and Tables

**Algorithm 1:**
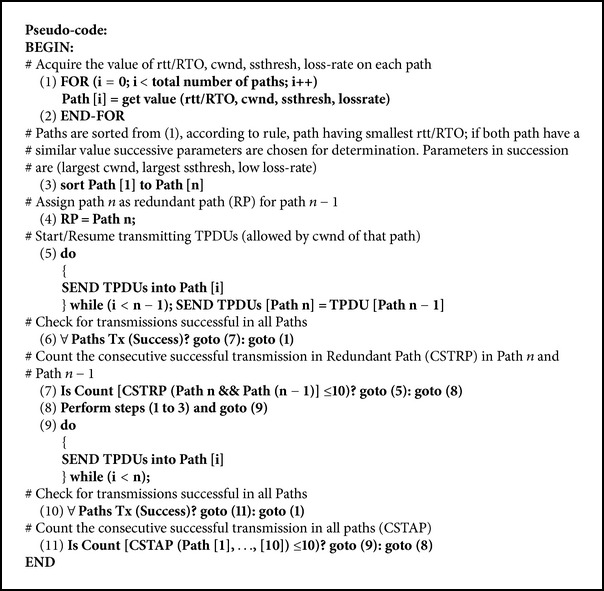
MSACMT-RTv2.

**Table 1:** Time taken for file transfer when rbuf = 128 KB.

Transfer file size (MB)	Cwnd = 2 MTU		
	Transfer time (s)		Percentage of time data transfer completed in advance
	MSACMT-RT	MSACMT-RTv2	
20	32.09	28.04	12.62%
30	45	39.08	13.16%
40	62.5	54.56	12.70%
50	70.42	60.5	14.09%
60	83	71.09	14.35%

**Table 2:** Overall data transfer time advanced.

Transfer file size = 60 MB; rbuf = 256 KB; cwnd = 2 MTU				
Each failure induced and lasting for 5 s				% of time data transfer completed in advance
Number of induced failures	Time at which failures are induced at _th (s)	Time taken for data transfer (s)		
		MSACMT-RT	MSACMT-RTv2	
1	10	55.02	48.05	14.58%
2	10, 20	61.3	53.26	15.09%
3	10, 20, 30	67.05	58.2	15.52%
4	10, 20, 30, 40	72.4	62.05	16.13%
5	10, 20, 30, 40, 50	78.09	66.8	16.77%

**Table 3:** Performance analysis for various file and buffer sizes.

Cases	Transfer file size (MB)	rbuf (KB)	Number of induced failures	% of time data transfer completed in advance
Nonfailure	40	128	0	12.70%
Failure				
Irregular intervals	40	256	3	10.34%
Regular intervals	60	256	1	14.58%
			2	15.09%
			3	15.52%
			4	16.13%
			5	16.77%

**Table 4:** Overall analysis during failure and nonfailure scenarios.

Transfer file size = 40 MB			
Cases	Number of induced failures	% of time data transfer completed in advance	
		rbuf = 128 KB	rbuf = 256 KB
Nonfailure	0	10.44%	13.05%
Failure			
Irregular intervals	3	07.13%	08.09%
Regular intervals	1	10.41%	11.93%
	2	10.92%	12.64%
	3	11.66%	13.35%
	4	12.47%	14.02%
	5	13.07%	14.94%

**Table 5:** MSACMT-RTv2 versus MSACMT-RT mean number of transmission.

Variant	Path 1 and Path 2 cross traffic %	Aggregate transmission		
		Path 1	Path 3	Path 3
MSACMT-RT	0	8698	8745	**7015**
MSACMT-RTv2		8607	8568	**8215**

MSACMT-RT	20	7192	7568	**9640**
MSACMT-RTv2		8536	8102	**10063**

MSACMT-RT	40	6978	7745	**9781**
MSACMT-RTv2		8201	7980	**11546**

MSACMT-RT	60	6645	7432	**10246**
MSACMT-RTv2		7984	7850	**13674**

MSACMT-RT	80	6430	7215	**10658**
MSACMT-RTv2		7438	7137	**16425**

MSACMT-RT	100	6108	6650	**11548**
MSACMT-RTv2		5540	5687	**20974**

**Table 6:** Asymmetric path delays—average throughput in kbps.

Loss	Nonfailure		Failure	
	MSACMT-RT	MSACMT-RTv2	MSACMT-RT	MSACMT-RTv2
Asymmetric				
Case 1	328.23	381.52	279.62	359.42
Case 2	316.21	368.34		
Symmetric	274.30	311.64	254.76	302.24
